# Correspondence

**Published:** 1901-09

**Authors:** J. P. Foster

**Affiliations:** Selby, South Dakota


### CORRESPONDENCE.

SELBY, SOUTH DAKOTA, July 25, 1901.

EDITOR JOURNAL OF COMPARATIVE MEDICINE, ETC. :

SIR : In replying to the communication from Dr. Howe, which appeared in the June number of the JOURNAL, I will say that in my opinion there is very little to answer, as I think that in my reply to Dr. Law, which appeared in the July number of the JOURNAL, I have touched quite thoroughly upon most of the points brought out by Dr. Howe. Dr. Howe, like his former teacher, Dr. Law, severely censures me for not telling "the truth ;" he also resembles the aforesaid teacher in publishing to the JOURNAL readers, over his own signature, the fact that he was a prize winner at Cornell. This was a case in which a non-prize winning Ontario graduate afterward became a prize winner at

Cornell. As I brought out in my reply to Drs. Law and Williams, the graduating class of 1897 at Cornell was composed of three members, viz., Drs. Howe, Ryder, and Weihe. It is my opinion that this is the same Dr. Ryder who, when fresh from his studies at Cornell, took the civil-service examination for a position in the Bureau of Animal Industry. Dr. Ryder made an average of either 80.5 per cent. or 81.5 per cent. in his examinations. Two Ontario graduates of 1884 and 1885 took the same examination. Neither of these Ontario men has ever attended a veterinary college since leaving Toronto. One of them got an average of 88 per cent., the other 88.25 per cent. I withhold the names of these Ontario graduates, as unlike Drs. Law and Howe they are not anxious to publish their achievements. In my reply to Drs. Law and Williams I attempted to argue the point to a logical conclusion that Dr. Weihe must of necessity have "put in" his two years previous to attending Cornell at a three-year school. I made this contention with the view of sustaining my position when I stated in my first article in the March number of the JOURNAL, in referring to Ontario graduates, that "they often at graduation capture first prize over those more fortunate students who have had the superior (?) advantage of having passed their whole period of professional study in the three-year school." I have since come into possession of the following facts: Dr. Weihe spent two years at Ames, Ia.; was then for about five years engaged in school teaching. From this position he went to the New York State Veterinary College (Cornell) and passed in one year. He was licensed by the New York State Board, but the State Board of Pennsylvania (in which State he had evidently started to practice) declined to accept the license granted him in New York State, and demanded that he should undergo an examination in Pennsylvania before he would be allowed to practice. This was followed by his removal from the State. As near as I can determine Dr. Weihe attended Ames after it had become a three-year school (January 1, 1888). My contention that Dr. Weihe "passed the whole of his period of professional study in the three-year school" therefore holds good. Dr. Howe, following the example of Dr. Law, distorts and misconstrues my statements to such an alarming extent that I wonder that he can "have the nerve" to advise me to "search for the truth, the whole truth, and nothing but the truth." In my reply to Drs. Law and Williams I think that I have explained what I meant by "whole period of study in the three-year school," but as this reply had not appeared at the time Dr. Howe wrote his communication (although the editor of the JOURNAL has been in possession of the copy of my "reply" since about May 15th) I excuse Dr. Howe for misunderstanding my meaning. Therefore, the argument advanced by Dr. Howe in the third paragraph of his article (the same paragraph in which he bashfully admits that he was a prize winner at Cornell) counts for naught.

Now about what Dr. Howe terms "comparing the two schools," and in the last two lines of his communication where he says "he will have no excuse for complaint against the New York State Veterinary College." The inference is that I have been "comparing" the Ontario Veterinary College with the New York State Veterinary College; also, that I have been "complaining" about the New York State Veterinary College. For one who is so ready to give advice in regard to truthfulness in others, Dr. Howe shows his inconsistency. The only time that I mentioned the New York State Veterinary College in the article which appeared in the March number of the JOURNAL, and the same that has brought forth replies from Drs. Law, Williams, and Howe, was as follows: After saying that Ontario graduates had taken first prize over students who had passed the whole of their professional study in the three-year school, I said "this has happened at the New York State Veterinary College, Ithaca, N. Y., and appears to be occurring with astonishing regularity for several years at McKillip" (allow me to digress at this point to say that since I wrote the article in question the same thing has again happened at McKillip. Dr. C. D. McGillvray, Ontario, 1900, received first prize and highest average for senior year (1900–1901) over "the more fortunate students," etc. I might also incidentally mention that the new secretary of McKillip, Dr. J. J. Millar, is an Ontario graduate of 1888). I also mentioned the fact that Cornell had an Ontario graduate of 1898 on the teaching staff (I use the word "teaching staff" for fear that I might be accused of an untruth if I said assistant professor).

These two instances constitute the whole grounds for my "complaining" and "comparing."

Dr. Howe's reference to the remarks I made concerning the gaudy catalogues of some of the colleges is almost too silly to notice, and were it not for the fact that even in this insignificant matter he misrepresents me when he says "as if red ink and embossing had anything to do with the advantages of a college," I should pay no attention to such remarks. In my original article, Dr. Howe, in speaking of the "red ink and embossing," do you find any place where I stated that the way to determine which was the best school was by the appearance of its catalogue?

Regarding the second paragraph of Dr. Howe's communication, I will say that it is almost a repetition of what I have said in my reply to Drs. Law and Williams; therefore, when Dr. Howe sees this reply I think that he will see that we have no argument on this particular point.

I wish it distinctly understood that this controversy was begun, so far as I am concerned, solely upon the defensive and not the offensive; in fact, Dr. Hoskins headed my first article "Defence of the Ontario Veterinary College." I suppose he chose this for want of a better title for the article, and I think he did very nicely. I had given the article no heading, but I think that the word "defence" is the right word in the right place. Were I to write my first communication over again I do not know how I could express myself any clearer, and, although I am now "up against" two "heavy weights" and a third "not *quite* so heavy," two of whom are "hinting" (some of said "hints" being pretty strong) that I do not have a proper regard for the truth, I will say that mere assertions count for little.

Drs. Law and Howe assert that I do not tell the truth; on the other hand, I am able to *prove* to any fair-minded person (and I think that I have done so through the columns of the JOURNAL that every statement I have made in regard to colleges and certain graduates of these colleges is *true*. Respectfully,

J. P. FOSTER.

